# The Book World of Medicine and Science

**Published:** 1893-11-11

**Authors:** 


### THE BOOK WORLD OF MEDICINE AND SCIENCE.

Handbook of Public Health and Demography. By Edward F. Willoughby, M.D.Lond., Diploma in State Medicine of the London University, and in Public Health of the Cambridge University. (London: Macmillan and Co., and New York.)

This is one of Macmillan's very excellent series of school class books, and we naturally turn to it with a strong prejudice in its favour. It is not, however, really a new book, for it is a much enlarged and carefully revised edition of Dr. Willoughby's "Principles of Hygiene. The number of works on this subject which are now issued is a strong proof of the greatly increased interest taken by the public in general in the important questions of personal and public health. It is one of the most encouraging "signs of the times." It is not long since hygiene was a very subsidiary feature in the curriculum of medical students, and was totally ignored by all other classes of students. This is being rapidly changed; we are witnessing a great diffusion of the knowledge of the laws of health, and of the conditions favourable to the spread of zymotic diseases, and|of the measures necessary for their prevention and extinction. In no case is it more true than in this that "knowledge is power." Such knowledge is of use to the individual for the regulation of his own life, to the head of a family to enable him to safeguard the physical welfare of his children, and to the citizen to initiate and promote measures of public safety. We gladly welcome this work, then, and hope it will find its way into multitudes of families and schools. Dr. Willoughby first deals with the "Health of the Man," and discusses in this section dietetics, clothing, habits, exercise, rest, &c.; then he passes on to consider the "Health of the House," and under this head he deals with the important questions of site, of ventilation and heating, drainage, and general construction. The "Health of the City" and the "Health of the People" are the appropriate themes of the 3rd and 4th chapters. Then follow chapters on Demography and Meterology, and a citation of the principal Acts relating to the pubilc health. All these subjects are treated concisely and clearly. Dr. Willoughby is thoroughly familiar with his subject, knows what he has to tell, and tells it in a simple and "understandable" form. While steering clear of too much detail, the author has made it a serious book, with plenty of information on the many points of importance in connection with personal and public hygiene. Thus the chemistry of foods is given without recourse to chemical formulæ, and the uses and preparation of foods are discussed in a really practical manner. And so on all through the book, a simplicity of statement is combined with practical teaching. The illustrations might with advantage have been more numerous—in all they are only thirty-nine—for they greatly help the reader, especially the young, and the untrained. It is a book well suited for schools, and if only all our youth were thoroughly versed in it, a long step forward in the path of hygienic progress would have been taken.

The Soil in Relation to Health. By H. A. Miers, M.A., F.G.S., F.C.S.; Assistant in the British Museum, late Examiner in the University of Cambridge; and R. Crosskey, M.A., D.P.H., Fellow of the British Institute of Public Health; Associate of the Society of Medical Officers of Health. With illustrations. (London: Macmillan and Co.)

Messrs. Miers annd Crosskey have compiled a useful little handbook of what may be called sanitary geology, which will enlighten many people as to what is desirable in selecting

THE BOOK WORLD OF MEDICINE AND SCIENCE—*continued.*

a site for a dwelling. To most the very names "ground air," and "ground water," will be wholly new, and it will be a revelation to some that these are quite as important to the health of a district as the air and water whose presence we recognize. The relation between certain kinds of soil and certain forms of disease has long been vaguely recognised, and many very unwarrantable theories have been based on it. The volume before us by its careful statement and studious moderation of tone will give just the prudent guidance that is required, without misleading people into the belief that they can shake off all diseases by merely shifting their camp. Special value attaches to the remarks on the various ways in which water may be contaminated, and the warning to householders not to trust to the "gravel soil" of the geological maps and the house-agent, without making sure that the vaunted gravel has not been removed, and replaced by "made soil" composed of road scrapings and all manner of filth; but the whole book is full of instruction at once scientific and practical.

Dissections Illustrated: A Graphic Handbook for Students of Human Anatomy. By C. Gordon Brodie, F.R.C.S., Senior Demonstrator of Anatomy, Middlesex Hospital Medical School; Assistant Surgeon North West London Hospital. With plates drawn and lithographed by Percy Highley. In four parts. Part II.—"The Lower Limb." (Whittaker and Co., London and New York.)

We are very glad to see the second part of this very useful anatomical atlas. There is no doubt that students find it a great advantage to read up their anatomy with accurate coloured representations of the parts before them. But most atlases of the kind have been issued in so expensive a form that the general run of medical students could only use them in the library of their medical school, and could not buy them. This atlas is issued at a price which puts it within the reach of nearly all medical students. The dissections drawn are those generally made, and the representations are very faithful and accurate. They are drawn on a rather larger scale in this part, and the change is a distinct improvement. The only criticism we would make is that the dissections follow the beaten track too closely; there is no attempt to show the relations of parts in other than the views generally obtained. But it is only fair to remember that the book is intended for students, and to enable them to recall the appearances they have previously observed in the dissecting-room. From this point of view the atlas is excellent.

The Scalpel is the name of a monthly magazine issued at the Medical College, Calcutta, for medical students studying in India. The September number begins with a brief editorial upon the disadvantages under which the Indian medical student labours. After commenting upon the enervating influence of the climate, it is candidly confessed that the standard of education is considerably inferior to that of Great Britain. It is stated, however, that much is being done in the way of improvement, and that in time it is hoped that the medical student in India will be as nearly on a footing with the student in Europe as circumstances will allow. The journal also contains a short article on post-partum hæmorrhage, reports of two cases, notes of football, a chapter of a serial tale, and a few odds and ends in the way of short humorous and unusual occurrences. Although it is admitted that the Indian student might be more energetic, the existence of a periodical is a sign of life and activity.

